# Genomic surveillance of SARS-CoV-2 in the state of Delaware reveals tremendous genomic diversity

**DOI:** 10.1371/journal.pone.0262573

**Published:** 2022-01-19

**Authors:** Karl R. Franke, Robert Isett, Alan Robbins, Carrie Paquette-Straub, Craig A. Shapiro, Mary M. Lee, Erin L. Crowgey

**Affiliations:** Research Department, Nemours Children’s Hospital Delaware, Wilmington, Delaware, United States of America; CEA, FRANCE

## Abstract

The use of next generation sequencing is critical for the surveillance of severe acute respiratory syndrome coronavirus 2, SARS-CoV-2, transmission, as single base mutations have been identified with differences in infectivity. A total of 1,459 high quality samples were collected, sequenced, and analyzed in the state of Delaware, a location that offers a unique perspective on transmission given its proximity to large international airports on the east coast. Pangolin and Nextclade were used to classify these sequences into 16 unique clades and 88 lineages. A total of 411 samples belonging to the Alpha 20I/501Y.V1 (B.1.1.7) strain of concern were identified, as well as one sample belonging to Beta 20H/501.V2 (B.1.351), thirteen belonging to Epsilon 20C/S:452R (B.1.427/B.1.429), two belonging to Delta 20A/S:478K (B.1.617.2), and 15 belonging to Gamma 20J/501Y.V3 (p.1). A total of 2217 unique coding mutations were observed with an average of 17.7 coding mutations per genome. These data paired with continued sample collection and sequencing will give a deeper understanding of the spread of SARS-CoV-2 strains within Delaware and its surrounding areas.

## Introduction

Since the severe acute respiratory syndrome coronavirus 2 (SARS-CoV-2) was first identified in Wuhan, China in December 2019 [1, 2], there have been over 198 million confirmed cases of COVID-19 and over 4 million deaths as of August 2021 (World Health Organization). The impact on the United States has been devasting for the healthcare industry and economic infrastructure. It has been noted that disease progression and outcomes for coronavirus disease (COVID) differ between adults and children, and that social determinants and ethnicity are linked to disparities in risk of disease and outcome.

The SARS-CoV-2 is a large RNA virus (30 kilobases) that contains 11 protein coding open reading frames (ORFs) including the 180 kDa capsid Spike protein; Spike facilitates binding to the receptor angiotensin converting enzyme 2 (ACE2) on host cells via the receptor-binding domain (RDB). After successful binding to ACE2, proteolytic cleavage of Spike allows for viral cellular involution and infection; given that antibodies targeting the RBD region can facilitate protection from viral infection by preventing this interaction, it is unsurprising that current vaccines use the Spike protein to stimulate an immune response [3].

Despite the goal of the national vaccine program to achieve a rate of vaccination sufficient for herd immunity [4], ~60% of the US population are fully vaccinated as of November 2021 (covid.cdc.gov). Furthermore, SARS-CoV-2 has a mutation rate of 1.12 * 10–3 mutations per site-year [5] and elevated rates of mutation have been reported in patients with prolonged cases and those with immunodeficiency or who are immunosuppressed [6]. As seen with other viruses, minor modifications of a viral genome can make vaccinations more or less effective, can be linked to disease outcome / severity, and can be useful for tracing transmission patterns. Most diagnostic tests are focused on the detection of the virus and do not yield information on the actual sequence of the viral genome. Following a positive diagnostic test for SARS-CoV-2 or a suspected false negative, next generation sequencing (NGS) is a powerful technique that can enable the rapid high-throughput sequencing of the SARS-Cov-2 RNA genome. This technique is proving to be important for understanding transmission patterns and potential host / viral interactions, which are analyses that cannot be conducted using the qPCR or CRISPR technique. Expanding SARS-CoV-2 genomic surveillance data in association with demographics and clinically relevant data will yield insights on the transmission patterns and pathophysiology of Covid-19.

The state of Delaware has fewer than a million residents but has had ~112,000 positive cases of SARS-CoV-2 infection and a total of 1,833 deaths (14.8 per 10,000 people) as of August 2021 (https://coronavirus.delaware.gov/). Previous studies have highlighted the genomic diversity of SARS-CoV-2 but few have focused on the complexity of that diversity within a single state. This analysis explores the sequencing results of 1,459 high quality SARS-CoV-2 positive samples collected in Delaware throughout the pandemic. These data reveal not only the specific SARS-CoV-2 strains infecting the population and the mutations they harbor, but also how those have changed over time as the genetic diversity of the viral genome has grown.

## Materials and methods

### Study approval

The study protocol was reviewed by the Nemours IRB (IRB #1688997) and was determined to be not human subjects research as only publicly available samples (PRJNA673096) were utilized or de-identified samples from Nemours were utilized.

### Nucleic acid extraction and COVID-19 screening of Nemours samples

Total nucleic acid was extracted from 140ul nasopharyngeal swabs via the Qiamp Viral RNA Mini kit according to the manufacturer’s protocol. 5ul of total nucleic acid was subjected to RT-PCR screening following the CDC’s 2019-Novel Coronavirus (2019-nCoV) Real-Time RT-PCR Diagnostic Panel guide. From March 2020 to May 2021 a total of 19,496 PCR COVID-19 tests were performed at Nemours Children’s Hospital Delaware. Of those, 489 were positive yielding a 2.5% positivity rate; samples with a CT value of less than 30 were selected randomly for sequencing. Sample collection dates are listed in S1 Table.

### Library preparation and sequencing of Nemours samples

100ng of total nucleic acid was used to prepare sequencing libraries with the Illumina RNA Prep with Enrichment kit and Illumina Respiratory Virus Oligo Panel. Library profiles and concentrations were evaluated on the Agilent 4150 TapeStation with the DNA1000 ScreenTape assay. Libraries with a size distribution of approximately 200bp to 700bp, with an average size of 350bp to 400bp, were pooled in equal mass and sequenced on an Illumina NextSeq 550 using PE74 reads with 5% PhiX spike-in. FASTQ files are available via NCBI accession PRJNA751858: SARS-CoV-2 Sequencing at Nemours Children’s Hospital Delaware.

### State of Delaware samples

FASTQ files were downloaded via NCBI accession PRJNA673096. Nasopharyngeal samples were de-identified and prepared using the ARTICv3 protocol and sequenced on the MiSeq (Illumina). Sample collection dates are listed in S1 Table.

### Data analysis

Sequencing data from libraries generated using Illumina’s RVOP V2 kit were analyzed using the DRAGEN RNA Pathogen Detection pipeline version 3.5.15 run in somatic mode with a MiniKraken2 (March 2020) reference database with dehosting enabled. Sequencing data from samples processed using the ARTICv3 protocol were analyzed using the Utah DoH ARTIC/Illumina Bioinformatic Workflow outlined on the CDC’s GitHub page (https://github.com/CDCgov/SARS-CoV-2_Sequencing/tree/master/protocols/BFX-UT_ARTIC_Illumina). Briefly, BWA MEM was used for read mapping, iVar was used for primer trimming, and samtools was used for consensus FASTA generation [7–9]. Any sequences with greater than 15% Ns were removed from the analysis. At least 10 reads were required to call any base pair. Consensus sequences were then submitted to NextClade and Pangolin for phylogenetic analysis and GISAID for mutation analysis. Protein sequences were aligned with Clustal Omega [10] and tree generation was performed via Geneious Prime 2021.1.1 using the Jukes-Cantor genetic distance model and the neighbor-joining tree build method.

## Results

### Lineage and phylogenetic analysis

**Fig 1:**
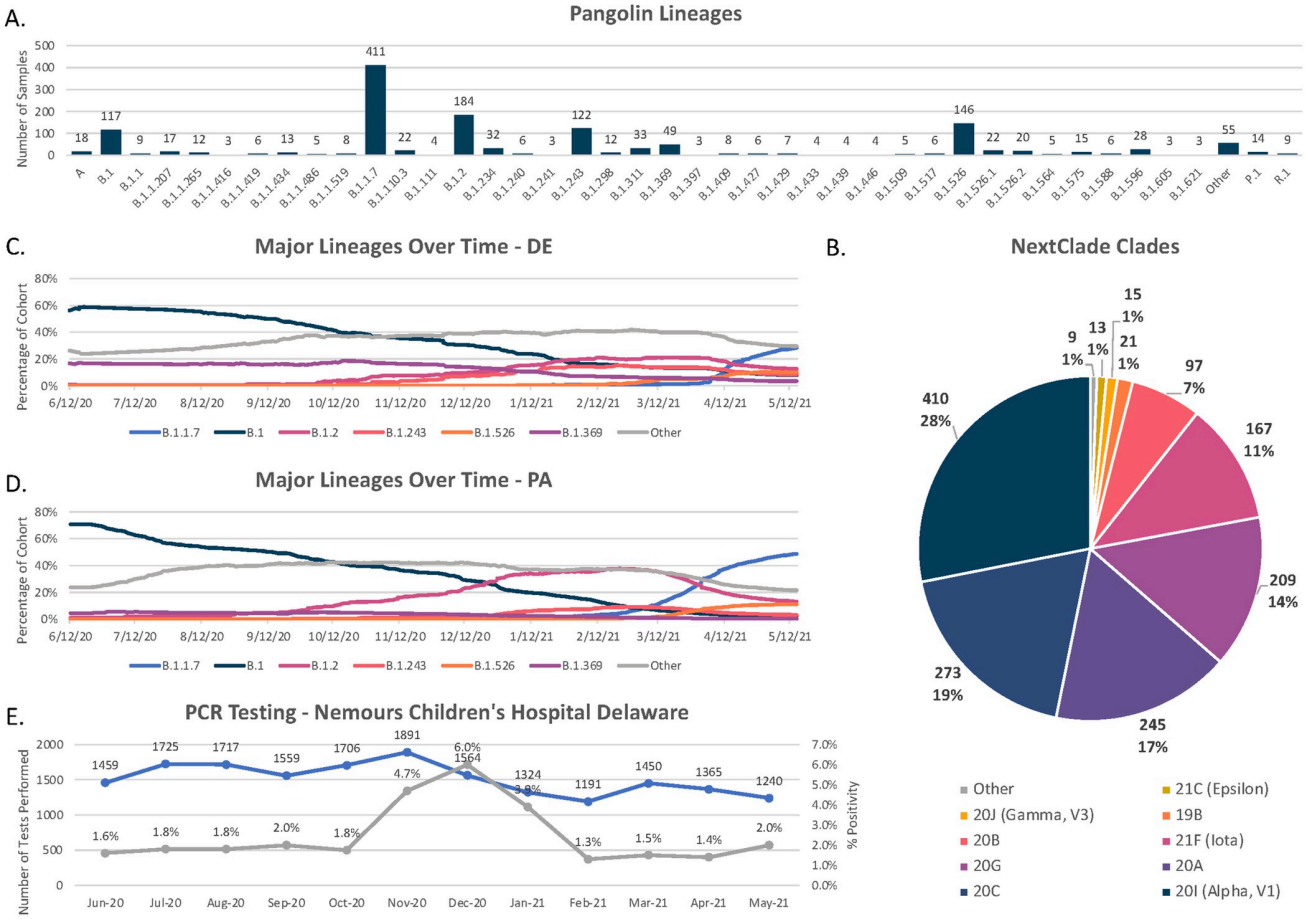
Lineage analysis of SARS-CoV2 genome from samples collected in the state of Delaware. Panel A: Pangolin lineages calculated from 1,459 samples. The x-axis is the lineage ID and the y-axis is the number of samples within that lineage. Panel B: The distribution of the NextClade Clades for all samples analyzed. Panel C: The major lineages over time in Delaware. The x-axis is the date, ranging from 6/20-5/21 and the y-axis is the percentage of that lineage in the total cohort analyzed. Panel D: The major lineages over time in Pennsylvania. Data Source—GISAID. Panel E: Results of COVID-19 PCR testing performed at Nemours Children’s Hospital Delaware.

Using the unique mutations within a viral genome, a lineage and phylogenetic analysis can be used to assign standardized lineages / strains independent of location and sample size. In total 1,459 samples were collected and analyzed in the state of Delaware. A lineage analysis was performed using both Nextclade’s clade assigner (S2 Table) and the Pangolin COVID-19 lineage assigner (S3 Table). Nextclade’s results contained 16 unique clades whereas Pangolin’s contained 88 lineages (Fig 1).

The predominant clade and lineage identified by both algorithms was the 20I-(Alpha, V1)/B.1.1.7 with 410 samples (28%) which first emerged from the UK in September 2020 and was reported to have increased transmissibility [11]. The second largest clade identified by Nextclade was the global 20C with 273 samples (19%); Pangolin categorized these into 31 unique lineages with most belonging to B.1, B.1.369, and B.1.311. Next was the global 20A clade which was assigned to 245 of samples (17%); these were assigned to 20 unique lineages by Pangolin, mostly the US based lineage B.1.243. Nextclade identified 209 samples as members of the 20G US specific clade originally identified by Pater et al. in January 2021 [12]. Pangolin classified most of these as the US strain B.1.2, except for 26 samples which were designated B.1.596. Plotting the proportion of the dominant lineages over time (Fig 1C) revealed the increase in diversity which began near the end of 2020; while this increase in diversity is correlated with an increase in COVID-19 testing positivity (Fig 1E), the holiday season and family gatherings are a more likely cause for the increased spread. Additionally, this analysis demonstrated that B.1.1.7 rapidly became the most dominant strain within a month starting in April 2021. To compare these results to those of a neighboring state, high quality COVID-19 sequences originating from Pennsylvania were downloaded from the GISAID database and classified via Pangolin (Fig 1D) and a similar trend was observed with B.1.1.7.

**Fig 2:**
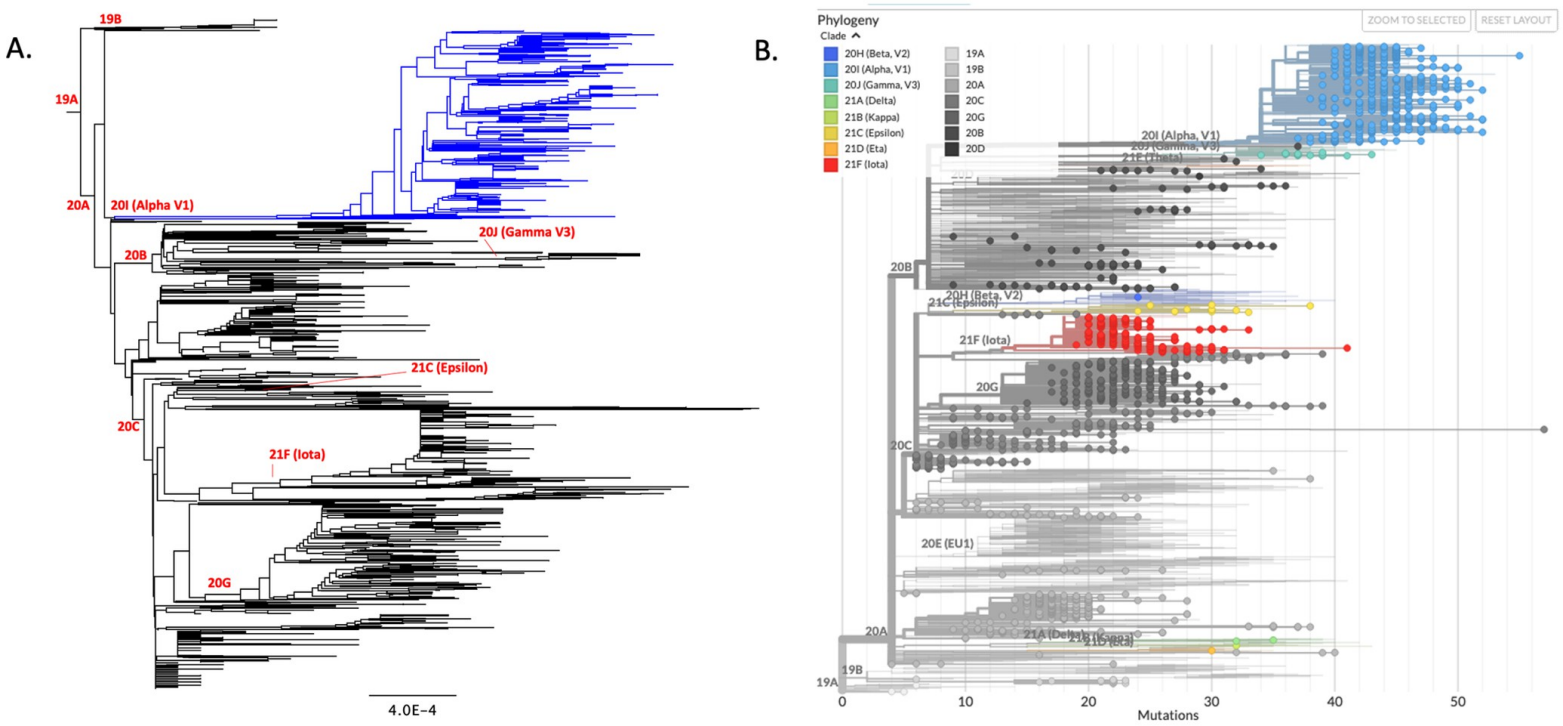
Phylogenetic analysis of SARS-CoV2 genome based on samples collected in the state of Delaware. Panel A: Phylogenetic tree of samples from the Delaware cohort only. Alpha/20I/B.1.1.7 highlighted in blue. Panel B: Nextclade phylogenetic tree of samples from the Delaware cohort superimposed on global sample set.

Phylogenetic analysis of protein sequences resulted in a tree with 2,917 nodes (Fig 2A). Most clades clustered as expected; 20A diverged from 19A, 20B and 20C diverged from 20A, and 21C-Epsiolon, 21F-Iota, and 20G all diverged from 20C. Based on literature it was expected to see 20I-Alpha diverge from 20B; however, this analysis demonstrated a divergence from 20A. Nextclade’s phylogenetic analysis combined their global database with our Delaware specific data (Fig 2B). This analysis highlighted several missing lineages, including 20D, 20E-EU1, and 20H-Beta clades, from the state.

### Mutation analysis

**Fig 3:**
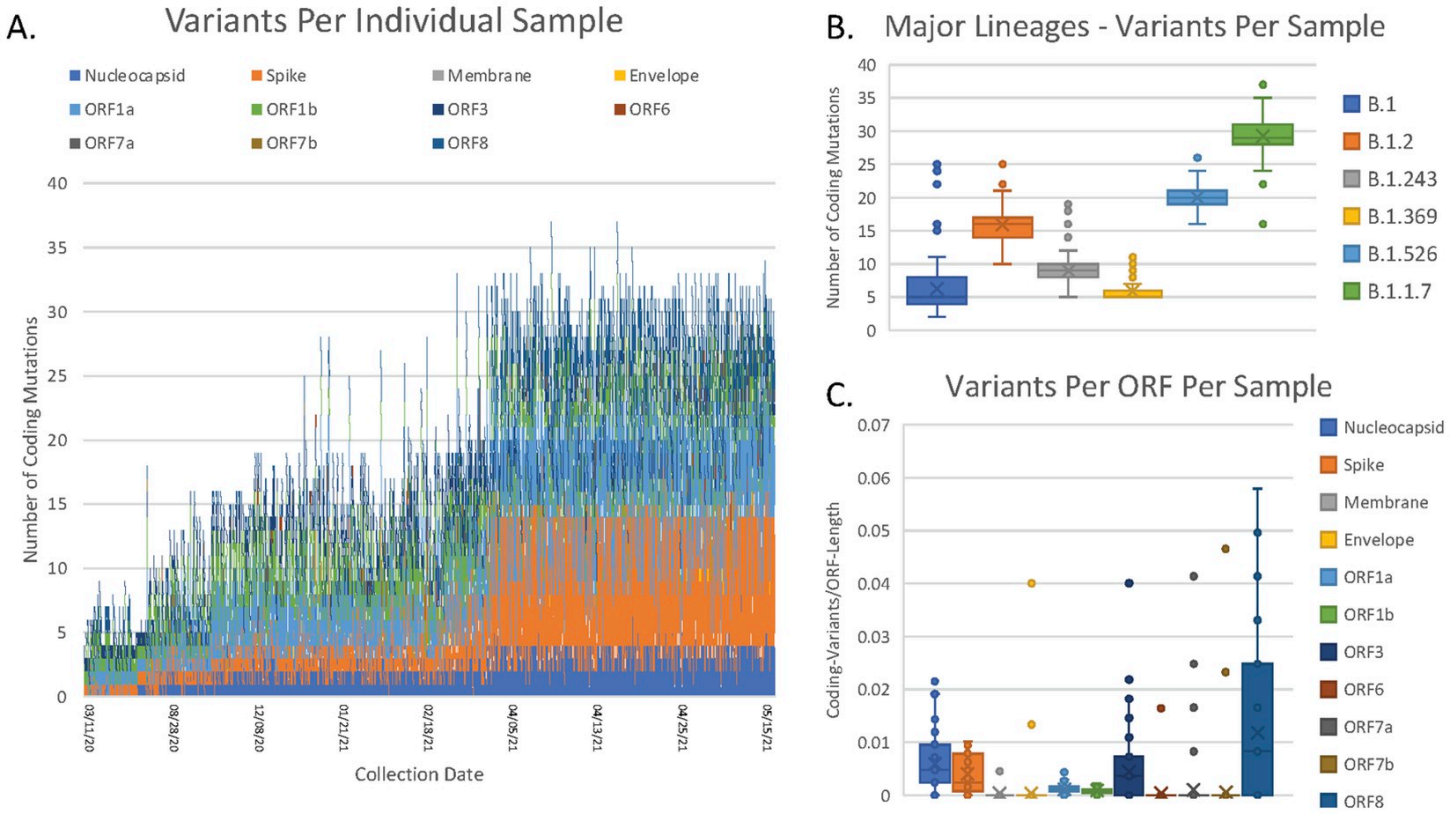
Analysis of coding variants identified in SARS-CoV2 genome. Panel A: Number of SARS-COV-2 coding variants detected per individual sample ordered by sample collection date. Please note that the X-axis is not linear due to increased testing performed later in the year. Panel B: Distribution of coding variants per sample broken down by major lineages. Panel C: Number of SARS-COV-2 coding variants per ORF normalized to ORF length.

The 1,459-sample Delaware based cohort had an average of 17.7 coding mutations per sample (range 2–37), and a median of 17 (S4 Table). A total of 2,217 unique coding mutations were observed, including 152 deletions, 14 insertions, and 26 premature stop codons. While 91% of the samples exhibited fewer than 30 coding variants, 131 samples had more with two presenting the maximum of 37 observed coding variants (Fig 3A). A clear trend can be observed with the average number of coding variants per sample increasing over time with a significant increase occurring near the end of March 2021 as highly mutated strains such as B.1.526 and B.1.1.7 became dominant. This led to far greater genetic diversity in the strains which emerged later in the pandemic as seen in Fig 3B. While the greatest number of variants were observed in ORF1a, this was unsurprising as it is the longest open reading frame and codes for a total of 10 proteins. When normalizing to length, it becomes clear that ORF1a is significantly less tolerant to mutations than ORF8, ORF3, and the nucleocapsid ORF (Fig 3C).

Four mutations were observed in more than 45% of samples sequenced (S5 Table). The top three found in the cohort were the globally dominant D614G in the spike protein (536 samples), P323L in NSP12 (529 samples), and Q57H in NS3 (348 samples) which were predicted to be stabilizing mutations [13]. The fourth mutation was T85I in NSP2 (310 samples); all samples exhibiting this mutation also had Q57H in NS3 which is consistent with previous observations [14]. Wang et al. characterized 12,754 SARS-CoV-2 genomes collected across the USA and found these four mutations to be the most prevalent; however, the percentage of samples in this Delaware cohort is slightly higher than what was observed across the USA.

There was significant deviation when comparing the most abundant mutations observed in these two cohorts beyond the top four. Wang et al. found R203K and G204R in the nucleocapsid to be the next most abundant mutations across the USA with 14% prevalence; however, these mutations were found at a much higher prevalence in the Delaware cohort at 36%, and four mutations (three deletions in NSP6, S106del, G107del, F108del, and P681H in Spike) were found at even higher prevalence at 43%.

**Fig 4:**
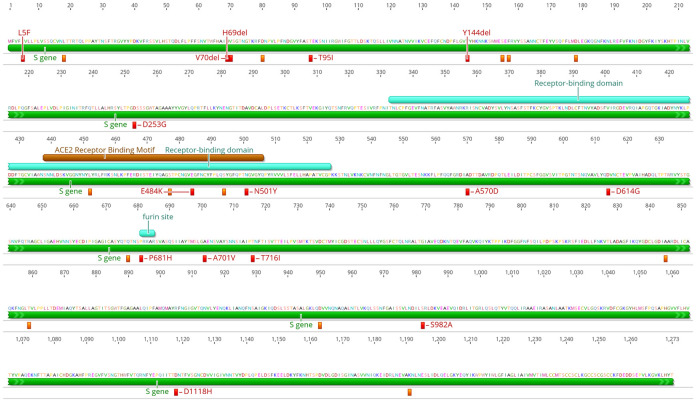
Schematic of the spike protein amino acids and location of spike protein mutations detected in the state of Delaware. All coding variants observed in more than 1% of samples in the Delaware cohort are pictured. Those variants seen in 10% or more samples are labeled in red.

A total of 354 unique coding mutations were observed in the spike protein specifically (Fig 4), with the five most prevalent appearing in at least 29% of samples: D614G (99%), P681H (39%), Y144del (31%), N501Y (29%), and T716I (29%). The P681H mutation has emerged spontaneously multiple times, as early as March 2020, in places such as Nigeria (GISAID Accession ID: EPI_ISL_729975), Hawaii [15], Israel [16], multiple times in New York State [17], and in the B.1.1.7 UK strain [11]; it is of particular interest due to its proximity to the furin cleavage site of importance for infection and transmission. The Y144del mutation is characteristic of B.1.1.7, but it has also emerged independently in other strains and was found to be present in 10 strains other than B.1.1.7 in this cohort. The N501Y mutation is characteristic of B.1.1.7, B.1.351, and P.1; this is of special concern as it has been shown to increase transmission efficiency via improved affinity of the spike protein for cellular receptors [18]. While this mutation was observed in the B.1.1.7 and P.1 samples of this cohort, it was also seen in three B.1.621 samples, a B.1.1 sample, and a B.1.324 sample.

## Discussion

While Delaware is a small state with a population of fewer than 1 million people, the lack of sales tax, its close proximity to Philadelphia and Baltimore, as well as the I95 corridor result in a significant amount of travel taking place through the state, especially in the northern areas around Wilmington. This Delaware cohort exhibited a significant increase in the frequency of a number of mutations (such as R203K and G204R in the nucleocapsid and S106del, G107del, and F108del in NSP6) compared to a similar profiling of strains across the entire country which examined samples through September 11^th^ 2020 [14]. This comparison is a good representation of how the landscape has changed over the past year. The three NSP6 deletions represent a 6nt deletion in NSP6 found in Alpha/20I/B.1.1.7 that has increased in prevalence with that strain, but has also emerged in other strains such as B.1.526; however, it is not known if this deletion plays any role in increased transmissibility. The two nucleocapsid mutations (R203K and G204R) have been shown to result in significant changes in the structural morphology of the protein [19] but there is no evidence that this results in increased transmissibility or severity of infection.

A number of the variants found in this cohort do have implications that are worthy of monitoring. The spike protein mutations P681H and Q677H are of particular interest due to their proximity to the furin cleavage site which has been proposed to enhance transmissibility of the virus via conformational change after cleavage [20]. The Q677H mutation disrupts the QTQTN consensus sequence adjacent to the cleavage site. This variant appears to be increasing in prevalence in the USA since late 2020, especially in the 20G strain, whereas previously it had been reported only sporadically outside the United States [21, 22]. The P681H mutation has occurred independently in multiple strains, but has not been associated with higher infection rates [16]. The A845S spike mutation was observed at the same frequency as Q677H; while little is known about its effects, it has been proposed to aid in the transmissibility of the Russian B.1.1.317 strain [23].

After normalizing for length, ORF8, ORF3, and the nucleocapsid ORF showed the greatest tolerance for mutations. Mutations that could lead to loss of function in ORF8 are of particular interest. SARS-CoV-2’s ability to persist without ORF8 function has been documented by multiple studies, and it is thought that this results in increased transmissibility and a milder but longer infection [24–26].

The CDC currently classifies four unique lineages as SARS-CoV-2 as either Variants of Concern or Variants Being Monitored as of November 2021: Alpha/20I/B.1.1.7 originating in the UK, Beta/20H/B.1.351 originating in South Africa, Delta/21A/B.1.617.2 originating in India, and Gamma/20J/P.1 originating in Japan/Brazil. All four of these lineages were identified within this cohort, however, only Alpha/20I/B.1.1.7 represented more than 1% of samples. The Delta/21A/B.1.617.2 lineage is currently of very high concern globally due to its increased transmissibility [27] and reduction in neutralization by post-vaccination sera due to the L452R spike protein mutation [28]. While only a single sample in this cohort was classified as Delta/21A/B.1.617.2, it took minimal time for the Alpha/20I/B.1.1.7 lineage to become the dominant strain in the state of Delaware (Fig 1C). While Pennsylvania is a much physically larger state than Delaware, B.1.1.7 needed only a slightly longer amount of time to become the dominate strain (Fig 1D) suggesting that this trend may not be limited to smaller communities.

A study based in Scotland recently showed that the Delta/21A/B.1.617.2 variant results in twice the risk of hospitalization compared to Alpha/20I/B.1.1.7 and a single vaccine dose is not sufficient protection [29]. At the time of this submission, only ~60% of Delaware residents have received both vaccine doses (https://coronavirus.delaware.gov; November 2021) putting the other half of the population at significant risk for hospitalization due to infection. These data showing how quickly Alpha/20I/B.1.1.7 was able to spread throughout Delaware coupled with what is known about Delta/21A/B.1.617.2 highlight the importance of this type of genomic surveillance. These efforts must continue so that hospitals are given ample warning to prepare for a future surge of Delta cases which is likely already be underway by the time this research is published. It should be noted that due to the massive volume of research being published on this topic, both in peer-reviewed and pre-print form, there are many other manuscripts relevant to this research which could not be cited here.

## Supporting information

S1 Table Sample collection dates. (XLSX)

S2 Table Nextclade results. (XLSX)

S3 Table Pangolin results. (XLSX)

S4 Table GISAID results. (XLSX)

S5 Table Mutation counts. (XLSX)
